# Emergent Functional Organization of Gut Microbiomes in Health and Diseases

**DOI:** 10.3390/biom14010005

**Published:** 2023-12-20

**Authors:** Marcello Seppi, Jacopo Pasqualini, Sonia Facchin, Edoardo Vincenzo Savarino, Samir Suweis

**Affiliations:** 1 Laboratory of Interdisciplinary Physics (LIPh), Physics and Astronomy Department, University of Padua, Via Marzolo 8, 35131 Padua, Italy; marcello.seppi@studenti.unipd.it (M.S.); jacopo.pasqualini@phd.unipd.it (J.P.); 2 Department of Surgery, Oncology and Gastroenterology (DiSCOG), University of Padua, Via Giustiniani 2, 35121 Padua, Italy; sonia.facchin@unipd.it (S.F.); edoardo.savarino@unipd.it (E.V.S.)

**Keywords:** metagenomics, dysbiosis, gut microbiomes, functional redundancy, emergent patterns, differential abundance analysis

## Abstract

Continuous and significant progress in sequencing technologies and bioinformatics pipelines has revolutionized our comprehension of microbial communities, especially for human microbiomes. However, most studies have focused on studying the taxonomic composition of the microbiomes and we are still not able to characterize dysbiosis and unveil the underlying ecological consequences. This study explores the emergent organization of functional abundances and correlations of gut microbiomes in health and disease. Leveraging metagenomic sequences, taxonomic and functional tables are constructed, enabling comparative analysis. First, we show that emergent taxonomic and functional patterns are not useful to characterize dysbiosis. Then, through differential abundance analyses applied to functions, we reveal distinct functional compositions in healthy versus unhealthy microbiomes. In addition, we inquire into the functional correlation structure, revealing significant differences between the healthy and unhealthy groups, which may significantly contribute to understanding dysbiosis. Our study demonstrates that scrutinizing the functional organization in the microbiome provides novel insights into the underlying state of the microbiome. The shared data structure underlying the functional and taxonomic compositions allows for a comprehensive macroecological examination. Our findings not only shed light on dysbiosis, but also underscore the importance of studying functional interrelationships for a nuanced understanding of the dynamics of the microbial community. This research proposes a novel approach, bridging the gap between microbial ecology and functional analyses, promising a deeper understanding of the intricate world of the gut microbiota and its implications for human health.

## 1. Introduction

In the realm of microbial ecology, our understanding of microbial communities has undergone a revolutionary transformation due to remarkable strides in sequencing techniques and bioinformatics pipelines [1,2,3]. These advances have opened an era where we can delve into the intricate tapestry of microbial community composition with unprecedented precision. Through methodologies like metabarcoding [4], we are now able to decipher taxonomic composition by sequencing distinct DNA segments, while metagenomic data enable us to quantify gene abundances within these intricate communities [5]. The novel dimension of metatranscriptomic data further allows for the quantification of gene expression levels, thereby enhancing our understanding of functional dynamics of the community [6]. Currently, elucidation of environmental chemistry is made possible through metabolomic data, enabling the reconstruction of the chemical environment in which these microbial communities thrive [7].

This robust expansion of our methodological arsenal has become the driving force behind research in microbial ecology, synergistically converging with computational biology to yield profound insights [8,9,10]. In this data-rich landscape, the focus of research is primarily on establishing qualitative associations between environmental factors and the composition of the microbial community. Typically pursued within a model-free framework, data analysis seeks to unravel patterns and relationships, often with limited theoretical underpinning. However, among this empirical wave, certain overarching trends and hypotheses emerge, transcending various ecosystems.

The variability inherent in community composition across temporal and spatial scales emerges as another hallmark, underscoring the dynamic nature of microbial assemblages [11,12].

Despite this variability, recent scientific investigations have focused on the statistical properties of abundance and diversity within microbial communities, pioneering the introduction of macroecology for microbiomes [13,14,15]. This approach characterizes these communities in terms of overarching macroscopic patterns, transcending the microcosmic variations observed at the taxonomic level. The essence of transitioning from microscopic variation, capturing the presence and abundance of specific taxa, to macroscopic patterns lies in the potential to unveil robust regularities that echo across diverse communities. In fact, some studies have advocated for this novel macroecological lens, underpinning the conceptual shift towards community characterization, also at the functional level [16,17,18].

However, it is unclear how much statistical characterization in sample taxonomy can illuminate and highlight the different ecological processes and mechanisms discriminating, for example, a diseased microbiome from a healthy one [19]. In particular, while the nuanced composition of microbial communities is inherently sculpted by ecological forces, it remains debatable whether the impact of these very forces is imprinted onto macroecological patterns.

This debate has older origins, as several works have highlighted how different ecological processes can lead to the same Species Abundance Distribution (SAD), a paradigmatic pattern studied since the latter half of the last century. Although the SAD has been used to support Neutral Theory, the veracity of this relationship is far from unequivocal [20,21,22]. In particular, it has been shown that, although healthy and unhealthy gut microbiomes display differences in species composition and abundance [23,24], their SADs have the same shape [25], and thus do not carry any signature of dysbiosis, defined as an ’imbalance’ in the gut microbiota community associated with disease.

Intriguingly, the stability of the functional composition, where gene groups are used as proxies, is on the one hand much higher than the taxonomic one [6,11], but at the same time strongly characterizes the type of microbial community.

Remarkably, functional and taxonomic compositions are founded on a shared underlying data structure, affording a unique avenue to leverage analogous strategies for characterizing the statistical landscape of these intricate assemblages. This parallel methodology, though methodological in essence, offers the promise of extending its scope beyond functional presence and abundance to encompass a macroecological scrutiny of functional correlations.

**Figure 1:**
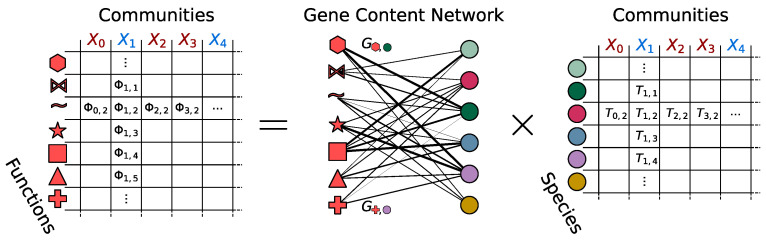
Visual representation of how functional tables $\Phi_{fs}$ are generated from a Genomic Content Network $G_{ft}$ and a Taxonomic Table $T_{ts}$ (where *f*, *s* and *t* are indexes, respectively, of the functions set, the samples set and the taxa set). In this figure, we pictorially represent functions with geometrical shapes, taxa with colored circles, and samples with indexed capital “X” symbols (blue when sampled from an healthy subject and red when sampled form an unhealthy one). While functional and taxonomic tables are represented as matrices, the GCN is represented as a network. The product symbol “×” represents standard matrix multiplication. In addition to this, the functional table is normalized to obtain a compositional dataset.

In this work, we want to make the first step in deciphering the statistical attributes of correlations among functional abundances, in particular by exploring if they carry signatures of dysbiosis. To achieve this goal, as shown in Figure 1, we will create taxonomic and functional tables from metagenomics sequences of healthy and unhealthy gut microbiomes, and we will investigate emergent functional macroecological patterns in both groups. We will extend differential abundance analysis to functions and gain insight into differences in functional compositions between healthy and unhealthy gut microbiomes. Eventually, we will show how different functions/functional categories interact among them and how studying such interactions is an effective way to characterize differences in functional organization between healthy and unhealthy microbiomes.

## 2. Materials and Methods

Our study is based on two different types of data. On the one hand, we have analyzed the metagenomic data of gut microbiomes obtained from two main studies [26,27], which collected stool samples from a series of control and clinical patients. On the other hand, we have exploited the Genomic Content Network (GCN) [28], obtained thanks to an annotated dataset [29], encoding information about the functional content of a certain set of taxa.

These two fundamental elements are processed and composed to produce a functional representation of each gut microbial community, as described below.

### 2.1. Taxonomic Dataset

Following Pasqualini et al. [25], we have obtained a metagenomic dataset with curated metadata, built from shotgun profiling of a set of 218 stool samples collected from clinical trials. Of these, 67 come from healthy individuals and 151 from patients affected by gastrointestinal tract diseases (i.e., Crohn’s disease, ulcerative colitis and inflammatory bowel syndrome). We address the first subset as healthy (H) and the second as unhealthy (U).

Based on pre-processed samples, we performed taxonomic profiling with the Kaiju [30] sequence alignment tool. We built the Kaiju index by collecting all UHGG proteomes and taxonomic files (nodes.dmp, names.dmp https://ftp.ebi.ac.uk/pub/databases/metagenomics/mgnify_genomes/human-gut/v2.0.1/kraken2_db_uhgg_v2.0.1/taxonomy/) [29] (accessed on 10 October 2022).

Proceeding in this way, we obtained a table having as column labels the IDs of the samples, and as row labels the IDs of the reference taxa set. Each entry in the table contains the number of reads from a specific sample that is assigned to a specific taxon.

From this intermediate result, we built a proper taxonomic dataset (TD). The TD is obtained by a three-step pipeline. First, we normalize the read counts assigned to each species with the genome length of such species, and further normalize each column to one to obtain compositional data [31]. Second, we set to zero all entries smaller than a certain threshold $\eta_{0}$, to reduce the presence of false positives. Third, we renormalize the columns in order to restore the compositionality of the dataset. In this way, we obtained a table (right table in Figure 1) in which each column consists of the relative abundance of microbial cells present within the sample (projected over the reference set of taxa).

We now discuss the three above steps in detail:

1. In order to obtain from a read count a cell count, we divided each number of reads by the genome length of the taxa to which it was assigned (each row is divided by the same genome length). Assuming that the probability of obtaining a read from a given genetic sequence is proportional to its length *L* (in the number of bases), by dividing the read counts by the genome length, we obtained a value that is proportional to the number of individual cells of taxa that was present in the sample [32]. In formulas, calling $T_{ts}$ the non-normalized dataset, we computed the normalized one, $T_{ts}$, as
  (1) $$T_{ts}=\frac{\frac{T_{ts}}{L_{t}}}{\sum_{t^{\prime }}\frac{T_{t^{\prime }s}}{L_{t^{\prime }}}}$$
  where *s* is an index over the set of samples, *t* and $t^{\prime }$ are indexes over the set of taxa and $L_{t}$ is the genome length of the taxon *t*.
2. The threshold was then applied to remove false positives [3]. Its value $\eta_{0}$ was chosen as the logarithmic flex point of the curve of the average local (α) diversity of $T_{ts}$, as a function of the threshold values [25]. Mathematically,
  (2) $$\eta_{0}=\underset{\eta }{\mathrm{argmin}}\frac{d\langle \alpha (\eta )\rangle }{d\log(\eta )},$$
  where 〈α(η)〉 is the average alpha diversity, calculated with the threshold η (specified below) over the relative abundances of the species (obtained from the previous step). The average was carried over the different samples. In other words, to compute 〈α(η)〉, for each sample, we counted the number of species whose compositional value was larger than η, and averaged this quantity over the different samples. After computing the threshold value $\eta_{0}$, we set all entries in the previous table with values smaller than $\eta_{0}$ to zero.
3. Finally, in order to restore the compositionality of the data (which was breached by the imposition of the threshold), we renormalized each column to one.

In this way, we obtained the taxonomic data set $T_{ts}$, which assigns the corresponding relative species abundance vector on the reference set of taxa to each sample.

### 2.2. Genomic Content Networks

A Genomic Content Network (GCN) [28] is a bipartite network where one set of nodes is made up by a catalog of genomes and the other by a chosen set of functions. The weight of a link in the network represents the number of times that a given function appears in the corresponding genome.

A Genomic Content Network (GCN) was built using a functionally annotated catalog of (metagenome-assembled) genomes [28]. Depending on the research question and bioinformatics expertise, several functional annotations can be chosen. The specific notion of the function used in this work is based on the eggNOG (evolutionary genealogy of genes: non-supervised orthologous groups) annotation [33], where each orthologous group is considered a function. Compared to other widely adopted annotations (such as PFAM [34] and KEGG [35]), eggNOG offers better coverage of UHGG (version 2.0.1 https://ftp.ebi.ac.uk/pub/databases/metagenomics/mgnify_genomes/human-gut/v2.0.1/) [29] (accessed on 10 October 2022), thus closely reflecting the functional repertoire of the considered genomes. Furthermore, these annotations employ 21 COG categories https://www.ncbi.nlm.nih.gov/research/cog/ (accessed on 10 October 2022) provided by the UHGG MAGs annotation [29] and available in the eggNOG database [33]. Such categories represent a coarse-grained functional level of gene clusters, allowing a more direct interpretation of the outcomes, as shown in the discussion section.

### 2.3. Functional Dataset

The functional dataset (FD) is obtained by matrix multiplication of the GCN with the TD, as shown in Figure 1.

In formula,

(3) $$\Phi_{fs}=\sum_{t\in \mathrm{taxa}}G_{ft}T_{ts}$$

where *f*, *t* and *s* are indexes of functions, taxa and samples, respectively, $\Phi_{fs}$ is the table of the FD, $G_{ft}$ is the adjacency matrix of the GCN and $T_{ts}$ is the table of the TD.

Note that, in order for this multiplication to be meaningful, the GCN and TD need to share the same taxonomic annotation. To achieve this, we used the GTDB annotation provided by the UHGG authors.

Each column of the TD can be interpreted as a vector in taxonomic space (relative species abundance vector). The GCN can thus be seen as a linear operator that connects the taxonomic space to the functional space and transforms, for each microbial community, relative species abundances into functional profiles, which are then normalized to obtain compositional data.

It may be argued that the same quantity can be obtained by directly aligning metagenomic sequences to a functional database. This is in principle correct and feasible, as the UHGG catalogue [29] contains a protein repository as well. However, such a protein catalog has many unannotated clusters, which makes a biological interpretation very difficult and induces inconsistencies between the taxonomy and the function classifications. Using the GCN, on the other hand, in addition to greatly speeding up the functional classification of samples, ensures that functional and taxonomic abundances are consistently linked, facilitating biological interpretation.

In this way, we obtained the functional dataset $\Phi_{fs}$, which assigns to each sample the corresponding relative functional abundance vector over the reference set of functions.

## 3. Results

Having generated the taxonomic and functional tables for both H and U groups, we evaluated whether specific signatures could be identified to characterize the dysbiosis. In this work, we focused on the “functional” profiles, showing only in some cases, for comparison, the taxonomic ones. In fact, taxonomic profiles have been already studied in several works, and recently on the same dataset in [25]. Confirming previous analyses, we found that looking at emergent patterns of taxonomic composition is not an enlightening way to find signatures of dysbiosis. We think, in fact, that due to a very strong functional redundancy, the point is not which microbes are present, but what their function is. Along this direction, we investigated the functional properties of the gut microbiome. We used the term “function”, although it is more correct to say “potential function”, as we were not looking at the metatranscriptome dataset, but only at the metagenomic data, and thus we could only reconstruct the possible functions/proteins that the different microbes can potentially express. In general, however, our approach can be applied also to metatranscriptome datasets.

**Figure 2:**
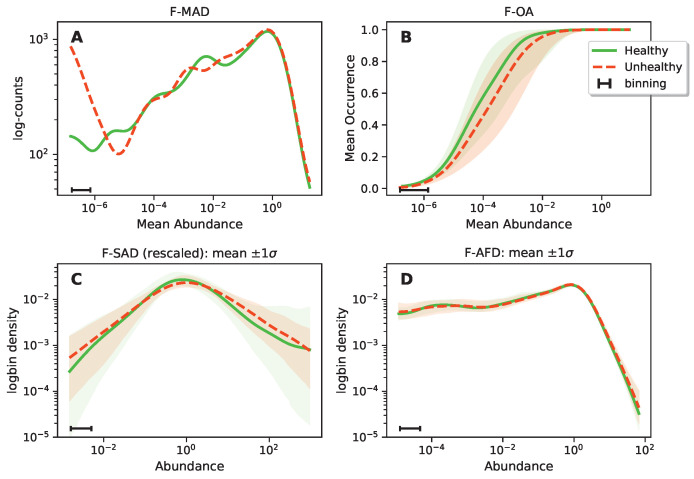
Main macro-ecological functional patterns: (**A**) Functional Mean Abundance Distribution (F-MAD). No significant difference between the healthy and the unhealthy patients over the abundant functions. (**B**) Functional Occurrence–Abundance profile (F-OA). The lines and their surrounding area represent the average $\pm \;\sigma_{\pm }$ of the values falling into the corresponding bin. (**C**) Functional Species Abundance Distribution (F-SAD). The F-SAD of each sample was rescaled to have zero mean and unit variance (in log units). The line represents the average among F-SADs obtained over different samples, while the shadowed area indicated the standard deviation (computed separately for the H and U subsets of SADs). (**D**) Functional Average Fluctuation Distribution (F-AFD). No rescaling was performed in this case on the single distributions. The F-AFDs are computed for the H and U subsets independently. The figure shows the averages among different F-AFDs and the standard deviation of the H and U subsets of AFDs. For both the F-SAD and F-ADF, we do not observe any relevant difference between the two classes (H and U). All the histograms are obtained as rolling windows, with a constant bin width in the logarithmic space.

We started by inspecting macro-ecological functional patterns as shown in Figure 2.

In Table 1, we present the main ecological patterns we investigated, their abbreviations and their definitions. Importantly, when the component of the system is an ecological species, then the pattern has a T prefix (Taxonomy), while when dealing with functions, it is indicated by an F prefix. All the ecological patterns are extracted separately for the H and U subsets. The first pattern we analyzed is the Functional Mean (relative) Abundance Distribution (F-MAD), which is the average relative abundance of a given function across H (green) or U (red) samples. We then computed the Functional Occurrence–Abundance profile (F-OA), giving us insight into the relation of the mean relative abundance of a function and its occurrence on the different H or U samples. As Figure 2B shows, functions that have an average relative abundance larger than $10^{-5}$ are present in almost all the samples. The Functional Species (relative) Abundance Distribution (F-SAD) instead is shown in Figure 2C. It describes the relative abundance of the different functions in a given H or U sample and typically displays a log-normal shape, similar to the SAD when looking at species instead of functions. The F-SAD of each sample was rescaled to have a zero mean and unit variance (in log units). Finally, Figure 2D shows the Functional Average Fluctuation Distribution (F-AFD), which gives the fluctuations of each function among H or U samples. No rescaling was performed in this case on the single-function distributions. The F-AFDs were computed independently with respect to the fluctuations over the H and U subsets, so that from each function we collected two AFDs.

From Figure 2, is quite evident that just comparing functional patterns did not lead to any clear signatures of different structures in H and U microbiomes. In particular, focusing on the more abundant functions, we did not find differences between H and U microbiomes in the F-MAD, F-OA or F-AFD. The F-SAD of U individuals had, on average, a larger number of both rare and abundant species, but these differences were not statistically significant if considering intra-group variability (shadowed regions). This “negative” result also holds if we look at the emergent macro-ecological patterns of species taxa (see [25]). Therefore, “first order” emergent macro-ecological and functional patterns in microbiomes in H and U groups are the same and do not characterize the “state” of the gut microbiome.

**Figure 3:**
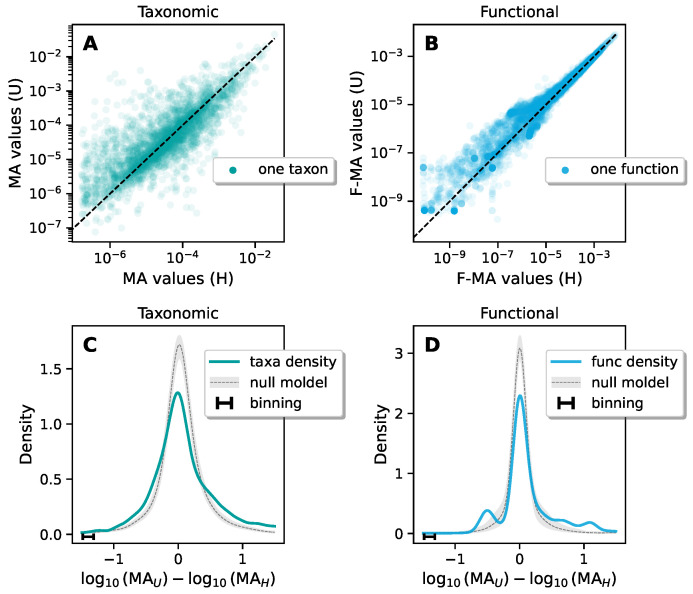
Differential abundance analysis of taxa/functions between the H and U sets. (**A**,**B**) For each taxon/function, we plot the mean abundance, as computed over the H and U sets, respectively, over the x and y axis. The dashed line indicates when H and U subsets have the same abundance. The taxa/functions above the line thus tend to be over-represented in U patients, while the ones below tend to be under-represented. (**C**,**D**) Histogram of logarithmic differences between the mean abundances of the taxa/functions over the U and H sets. The gray region represents the null model (average $\pm \;\sigma_{\pm }$) obtained by randomly redistributing the H/U labels over the samples. Both the taxonomic and functional plots display an enrichment of the tails. In particular, in the functional plot, we observe the emergence of secondary peaks.

Nevertheless, we investigated possible differences in the abundance of single functions or taxa in H and U gut microbiomes (Figure 3). This approach is well established when looking at taxa and it is known as a Differential Abundances Analysis (DAA). A multitude of DAA tools have been introduced specifically tailored to address sampling and compositional effects [36], and they play a crucial role in singling out microbial species characterizing dysbiosis.

In the upper sub-figures Figure 3A,B each point corresponds to a specific component. The x and y projections correspond, respectively, to the H and U expressions of such components, and the expression degree of a component is defined simply as its mean abundance (over the H or U subsets). In the lower sub-figures Figure 3C,D, we build a histogram of the differential logarithmic expressions of the components between the U and H sets.

In formulas, if we call $MA_{i}^{\left(H\right)}$ the mean abundance of the *i* component over the H subset and $MA_{i}^{\left(U\right)}$ the mean abundance of the *i* component over the U subset, the logarithmic differential expression of component *i*, $\delta_{i}$, is defined simply as

(4) $$\delta_{i}\equiv \log_{10}\left(MA_{i}^{\left(U\right)}\right)-\log_{10}\left(MA_{i}^{\left(H\right)}\right).$$

These quantities were then collected into a histogram. The components that fall into the right part of the histogram are the ones that have a tendency to be enriched in going from H to U. The components that fall into the left part, instead, are the ones that tend to be depleted.

To be more quantitative, we compared such differential abundances with proper null models. The null model consisted of 150 independent realizations in which we randomized the H/U attributes of the samples, keeping intact the cardinality of the two sets. This null model allows us to contrast between the condition in which we confront two random sets and the condition in which we confront the H and U sets. An anomaly with respect to this null model is therefore to be attributed to the specificity of the choice of H and U as subsets. The null model is represented in Figure 3 as the (point by point) average $\pm \;\sigma_{\pm }$ of the values obtained in the different realizations, where $\sigma_{+}$ is the root mean square of the positive fluctuations (only from above average values), and $\sigma_{-}$ is the root mean square of the negative fluctuations (only from below average values). We therefore expect random realizations to fall within the highlighted area.

We found that for taxonomic DAA, in spite of the presence of tail enrichment, we could not identify a set of particular species that are responsible for such an effect (see Figure 3C). On the other hand, for functional DAA (FDAA), we could identify a group of functions whose abundances differ significantly from the null model (see bumps in Figure 3D). In other words, in taxonomic DAA, we had a specificity issue, and thus we could not exploit DAA to understand the ecological processes behind the dysbiosis. For functions, instead, we could recognize some functional categories characterizing the enrichment or depletion.

Thus, to further test such a hypothesis, we explored two different computational approaches for detecting the main functional groups responsible for dysbiosis. In both cases, we constructed the “discriminant direction” vector in functional space. With *discriminant direction* we intend, loosely speaking, the notion of a direction (in functional space) along which one passes from the H to the U condition. We implemented this concept in two specific ways, which we refer to as the differential mean abundance analysis method (*MA method*) [36] and Support Vector Machine method (*SVM method*) [37].

The MA method defines the discriminant direction as the difference between the F-MA of the U functions and the F-MA of the H functions.

$$\xi_{i}\equiv MA_{i}^{\left(U\right)}-MA_{i}^{\left(H\right)}.$$

The discriminant direction $\xi_{i}$ is therefore a vector in a (high-dimensional) functional space.

The SVM method instead defines the discriminant direction $\xi_{i}$ as the tangent vector to the optimum dividing hyperplane between the H/U-labelled data in functional space, as defined by a standard SVM procedure [37] (choosing the orientation that leads from the H-classified to the U-classified subspace).

**Figure 4:**
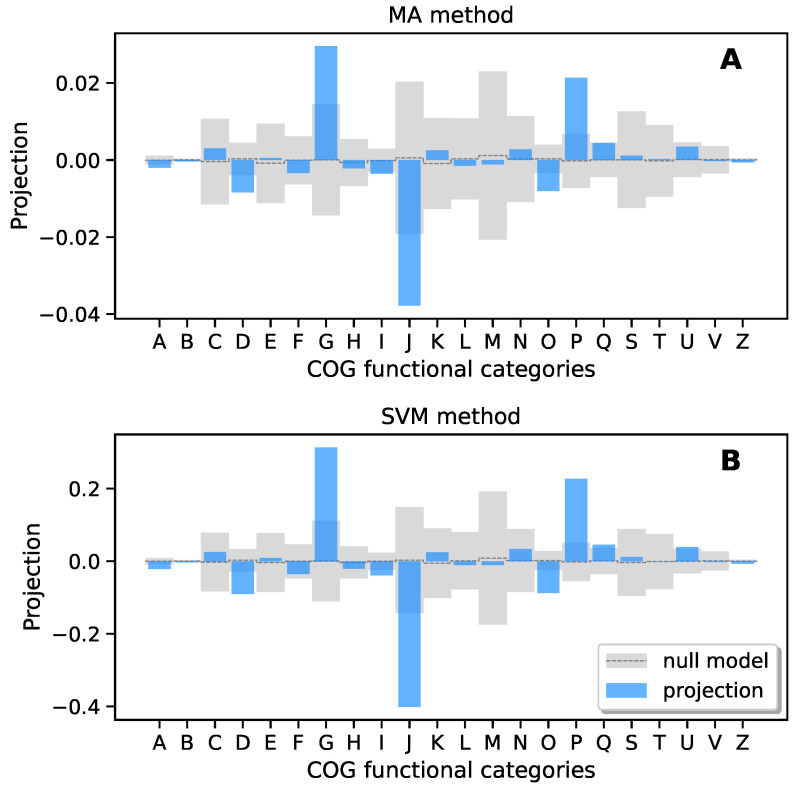
Discriminant direction within the functional space (ideally leading from the H region to the U region) projected on COG functional groups. (**A**) MA method. The discriminant direction vector is defined as the difference between the F-MA vector of the unhealthy and the F-MA vector of the healthy. (**B**) Support vector machine (SVM) method. The discriminant direction in the functional space is defined by the tangent vector of the optimal dividing plane between the H and the U, as defined by a standard support vector machine procedure. The two procedures produce almost identical results, showing a significant enrichment in categories G and P, and a significant depletion in categories D, J and O.

We further project the $\xi_{i}$ vectors over the COG functional groups [38], which constitute a low-dimensional set of general functional categories which can be directly interpreted. In this way, we obtain the enrichment/depletion profiles shown in Figure 4.

The null model is defined again as a set of 150 repetitions of independent random redistributions of the H/U attributes between the samples. Over each realization, we repeated the same analysis (computation of $\xi_{i}$, projections over categories and normalization), summarizing the results as the average ±σ.

Consistently, in both approaches (MA and SVM methods), we may recognize that some functional categories deviate significantly from the null model. In particular, we recognized three under-represented functional groups (D—Cell cycle control and mitosis, J—Translation and O—Post translational modification, protein turnover, Chaperone functions) and two over-represented functional groups (G—carbohydrate metabolism and transport and P—inorganic ion transport and metabolism) in U with respect to H samples.

In the last part of this work, we studied the correlations between functions/functional categories in H and U samples.

We performed a comparative analysis of the correlation structure of the system along two “axes”: the taxonomic/functional difference, and the H/U difference. So, on one hand, we observe how the taxonomic correlation structure differs from the functional one, and on the other hand, we determine how the H or U conditions impact the correlation structure (both in taxonomic and functional settings). Significantly, we also investigated the connection between correlation and abundance, and how this connection differed in the taxonomic and functional settings. Are couples of components (either two taxa or two functions) with big values of mean abundance more likely to be correlated?

**Figure 5:**
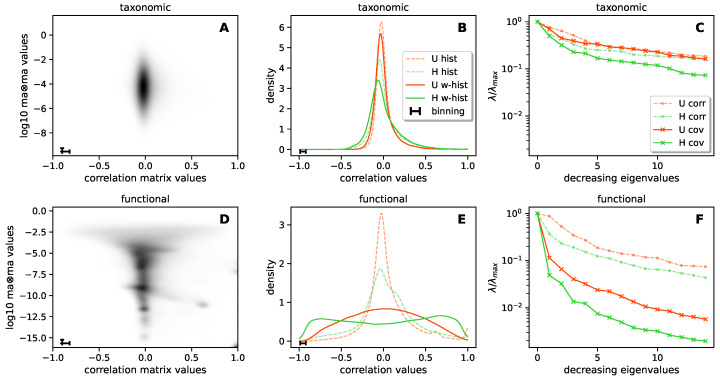
Correlation structure, comparison of H and U subsets from a taxonomic and functional point of view: (**A**,**D**) 2D histogram. To each couple of taxa/functions we associate (i) the corresponding entry of the taxa–taxa/function–function correlation matrix and (ii) the product of the mean abundances of the two taxa/functions. We then construct a 2D histogram of such quantities as realized by iterating over all the couples of taxa/functions. From a taxonomic point of view, we observe an almost complete independence between the two quantities. From a functional point of view, we observe a strong connection. (**B**,**E**) Histograms of the values of the taxa–taxa/function–function correlation matrix. The green/red values pertain to the correlation within H/U subsets. The dashed lines are realized as standard histograms. The solid lines are realized as weighted histograms; the weight is the product of the mean abundances of each couple of taxa/functions. We observe that the weighted histograms reveal a much stronger correlation structure with respect to the standard ones; in both cases, the H subset displays a stronger correlation structure with respect to the U subset. Such a difference is much stronger in the functional representation of the system. (**C**,**F**) Decay profile of the eigenvalues of correlation and covariance matrices, both for H and U subsets. We observe that the H profiles decay faster than the U profiles and the covariance profiles decay faster than the correlation profiles. In the functional representation, these effects are once again much stronger.

To answer this question, we computed the 2D histograms displayed in Figure 5A,D. Such histograms were produced using the values of the correlation matrix and the values of the couple abundance matrix (defined by the couple products between the mean abundances (MAs) of the two components). In the formulas, $C_{ij}$ is the Pearson’s correlation coefficient between components *i* and *j*; $A_{ij}=MA_{i}\cdot MA_{j}$ is the product of the mean abundances components *i* and *j*. We can therefore associate a value $C_{ij}$ and a value $A_{ij}$ with each couple (i,j), and we compute a bi-dimensional histogram of such values. In this part of the analysis, we did not distinguish between H and U. We found that the most abundant functions were commonly strongly correlated or anti-correlated, highlighting a correlation structure that was instead absent in the taxa space.

The correlation profiles in Figure 5B,E were then computed by histogramming the values of $C_{ij}$ in two ways. The standard (*even*) histogram is computed by counting each value with the same weight (one). We may also assign a different weight to each value, so that some values are more important than others in the overall counting. In this case, we talk about a *weighted* histogram. We produced the histograms of the correlation matrix values $C_{ij}$ in both even and weighted ways, choosing the values $A_{ij}$ as weights. A weighted histogram is a way to give proportionally more importance to the couples which contain more abundant components. We thus looked at the obtained correlation profiles in both taxa and functions and in both H and U microbiomes. In all cases, we found that H samples were characterized by a larger number of taxa/functions that were strongly correlated or anti-correlated. For the functional correlations, we could observe that such functions were also on average the most represented (see Figure 5D), i.e., highly abundant functions were those displaying higher correlations. Therefore, by weighting the correlation function by the species average abundances, we were able to further highlight the difference between H and U samples in their functional correlation structure.

Such differences were clearly reflected in the correlation and covariance matrix eigenvalue decay curve for H and U samples in both the taxa and functional spaces, as shown in Figure 5C,F. Recall that each eigenvalue measures the variance of the dataset along the direction of the corresponding eigenvector in a unit system in which each direction in the original basis set is rescaled to have unit variance. The covariance matrix instead does not contain such a rescaling, and the eigenvalues measure the variance along the direction of the eigenvectors in the original units of the dataset. Loosely speaking, we can thus consider the correlation matrix to define a notion of effective dimensionality according to *relative* fluctuations, while the covariance matrix defines it according to *absolute* fluctuations. These results allows us to recognize how different factors impact the effective dimension of the data by comparing the decay rate of the eigenvalue spectrum in the different cases. By comparing the U results with the H results, we can observe how the H condition is associated with a smaller effective dimensionality; by comparing the correlation matrix profiles with the covariance matrix profiles, we can observe how considering the difference in scales of the abundances leads to a smaller effective dimensionality. Finally, by comparing the results in Figure 5C,F (drawn on the same scale), we can observe how the previous effects are strongly amplified from a functional prospective.

In other words, significant differences between H and U microbiomes emerge when looking at patterns of correlations from a functional prospective.

## 4. Discussion and Conclusions

In this work, we have studied the emergent functional organization in both healthy and unhealthy gut microbiomes. In particular, we have investigated if and which functional unbalance can characterize dysbiosis.

First, we have shown that global emergent functional patterns, inspired by macro-ecological laws in ecology [15], do not characterize the particular state of gut microbiomes, and thus cannot be directly exploited to find specific signatures of dysbiosis. On the other hand, by employing a functional abundance analysis and a support vector machine algorithm, we have found distinct signals of functional categories that are unbalanced between healthy and unhealthy gut microbiomes. Furthermore, an analysis of the correlation structure of taxa and functions showed the existence of a strong relation between functional correlation and functional abundance, revealing the existence of a core of strongly correlated functions, which has no analogous feature in the taxonomic perspective, and that the correlations within such core are significantly disrupted in dysbiosis.

Our results consistently demonstrated significant alterations in the abundance of functional categories in unhealthy gut microbiomes (when compared to their healthy counterparts), particularly within the COG functional categories D (cell cycle control, cell division and chromosome partitioning), J (translation, ribosomal structure and biogenesis), O (post-translational modification, protein turnover, chaperones), G (carbohydrate metabolism and transport) and P (inorganic ion transport and metabolism).

The under-representation of functions related to cell cycle control, cell division and chromosome partitioning (D) in unhealthy gut microbiomes indicates that microbial cells may replicate in an abnormal way in the diseased hosts. This abnormal growth may contribute to the community composition unbalance observed in dysbiosis. Of course, the causes of such altered cell division processes remain elusive and may depend on different factors (gut inflammation, changes in the environment and thus bacterial–host interactions, or changes between species interaction, e.g., from cross-feeding). Similarly, the under-representation of translation-related functions (J) in unhealthy gut microbiomes may be of biological significance. A reduced abundance of translation-related functions may imply a decrease in protein production, which could impact various physiological processes in the host. This observation may be associated with the metabolic alterations commonly seen in gastrointestinal diseases, which often manifest as malabsorption and nutritional deficiencies [39]. Post-translational modifications, protein turnover and chaperone functions (O) are intricately linked to protein stability and functions. The under-representation of O functions in unhealthy gut microbiomes suggests that the microbiota may exhibit a reduced capacity to ensure proper protein folding, stability and turnover. This could lead to an accumulation of misfolded or dysfunctional proteins, potentially contributing to cellular stress and inflammation.

The over-representation of carbohydrate metabolism and transport functions (G) and inorganic ion transport and metabolism functions (P) in unhealthy gut microbiomes may be interpreted as possibly associated with the metabolic adaptations of gut microbiota in response to disease states. Over-representation of carbohydrate metabolism functions may indicate an increased utilization of available carbohydrates, possibly as an energy source. This may be linked to the characteristic shifts in dietary preferences and nutrient availability seen in patients with gastrointestinal diseases [40].

The over-expression of inorganic ion transport and metabolism functions (P), particularly related to iron (Fe) and copper (Cu), is noteworthy in the context of gastrointestinal diseases. Indeed, correlations between IBD and host iron deficiency and alterations in Fe metabolism are commonly observed [41]. Research on the impact of iron supplementation on gut microbiota has yielded diverse results, making generalizations difficult. The chemical form of iron used can also influence the bacterial composition, and the effects of iron supplementation do not always directly oppose those of iron deficiency. Heme iron is more readily absorbed by both bacteria and humans compared to non-heme iron, as it is influenced by the composition of the food matrix and the physico-chemical conditions in the digestive tract. Non-heme iron in many food matrices is bound to inhibitors like polyphenols, fibers or phytates. Bacterial enzymes can break down these inhibitors, improving iron absorption [42]. The over-representation of P functions may thus reflect the adaptive response of the microbiota to address the unbalance due to loss in functions correlated with cell cycle control and increased demand for iron, possibly due to chronic inflammation or blood loss in the gastrointestinal tract.

We therefore think that the observed under-expression of cell cycle control (D), translation (J) and protein modification functions (O) may contribute to tissue damage and dysfunction, while the over-expression of carbohydrates (G) and ion (P) metabolism functions may represent metabolic adaptations to disease-related challenges.

Still, one needs to consider that the over/under-representation of functional categories may be affected by significant bioinformatic noise in the attribution of the functions to the categories. In order to assess the stability of our results, we performed the same analysis with the updated functional annotations directly provided by the COGs website [38,43] (instead of the previously used annotations provided by UHGGv2.0.1). The results are generally quite consistent, but report a reduced discrepancy in the P category, while other previously non-anomalous or non-present categories show a relevant discrepancy (L replication, recombination and repair; X mobilome: prophages, transposons). This may indicate the need to distinguish between strong signals (such as those observed for the alterations of G and J) and weak signals (such as those observed for the alterations of the other categories).

Eventually, in the analysis of the correlation structure of the functional abundances among different patients, we found evidence of a core of abundant and correlated functions. This may be interpreted as a way of representing (with a soft constraint) the allowed functional configurations of a healthy microbiota. Remarkably, in the unhealthy subset, we observed a much weaker (core) correlation structure, as almost all the correlations higher than 0.9 and lower than −0.9 were suppressed. This indicates that the microbiota of unhealthy patients are subjected to a weaker constraint on the functional organization, or are subjected to many inhomogeneous organizing principles. This means that in dysbiosis, the coordination (or anti-coordination) of functions is somehow disrupted, even when the general macroecological patterns are conserved. A weakening of the correlation structure may also be observed in the taxonomic framework; here, however, this tendency is masked by the high degree of functional redundancy.

In particular, we have also found that if we project such functions onto the space of COG functional categories and study the correlations between functional categories, unhealthy microbiomes, differently from the healthy ones, do not display any relevant negative correlation, typically associated with stabilizing negative feedback loops.

In summary, our study provides valuable insights into the functional changes in gut microbiomes associated with gastrointestinal diseases. We have shown the usefulness of functional differential abundance analyses and the study of correlation patterns in highlighting such changes, and particularly that dysbiosis is associated with a loss of correlation between the core functions. A natural and important direction is to combine such an analysis with a similar one, but using meta-transcriptomic datasets. This would allow for an effective study of the actual expression of functions for the specific class of microbiomes, together with the potential activity, as measured through metagenomics. These findings underscore the intricate relationship between gut microbiota and host health in the context of gastrointestinal diseases and emphasize the potential utility of multi-omics techniques (and related statistical analyses) in investigations of dysbiosis and related disease mechanisms, as well as in the definition of potential biomarkers for the detection of diseases.

## Figures and Tables

**Table 1:** Description of the macroecological patterns. All of the following definitions can be applied both to the taxonomic and the functional table, and in general to any component system.

Macroecological Pattern	Abbreviation	Definition
Abundance Fluctuation Distribution	AFD	Distribution of the abundances of a given component (*i*) among different samples.
Species Abundance Distribution	SAD	Distribution of the abundances of the components within a given sample.
Mean Abundance Distribution	MAD	Distribution of the mean abundances (among samples) of the components.
Occurrence	O	Fraction of samples in which a given component (*i*) is present.

## Data Availability

All the data used in this study are available from previous works [27,44] (SRA BioProjects PRJNA400072, PRJNA398089). Metadata collection and processing are discussed in [25]. The code for metagenomic preprocessing, sequence alignment and GCN building can be found at https://github.com/jacopopasqualini/MetaGym (accessed on 10 October 2022). The rest of the code may be found at https://github.com/MarcelloSeppi/Gut-micro-org-2023 (accessed on 14 December 2023).

## Abbreviations

The following abbreviations are used in this manuscript:

GCN	Genomic Content Network
TD	Taxonomic Dataset
FD	Functional Dataset
H	Healthy
U	Unhealthy
(F-)MAD	(Functional-)Mean Abundance Distribution
